# *Toxoplasma gondii* Elongation Factor 1-Alpha (TgEF-1α) Is a Novel Vaccine Candidate Antigen against Toxoplasmosis

**DOI:** 10.3389/fmicb.2017.00168

**Published:** 2017-02-13

**Authors:** Shuai Wang, Zhenchao Zhang, Yujian Wang, Javaid A. Gadahi, Lixin Xu, Ruofeng Yan, Xiaokai Song, Xiangrui Li

**Affiliations:** ^1^College of Veterinary Medicine, Nanjing Agricultural UniversityNanjing, China; ^2^School of Basic Medical Sciences, Xinxiang Medical UniversityXinxiang, China

**Keywords:** *Toxoplasma gondii*, elongation factor 1-alpha, host cell invasion, protective immunity, vaccine

## Abstract

*Toxoplasma gondii* (*T. gondii*) is an obligate intracellular parasite which can infect almost all warm-blood animals, leading to toxoplasmosis. Screening and discovery of an effective vaccine candidate or new drug target is crucial for the control of this disease. In this study, the recombinant *T. gondii* elongation factor 1-alpha (rTgEF-1α) was successfully expressed in in *Escherichia coli.* Passive immunization of mice with anti-rTgEF-1α polyclonal antibody following challenge with a lethal dose of tachyzoites significantly increased the survival time compared with PBS control group. The survival time of mice challenged with tachyzoites pretreated with anti-rTgEF-1α PcAb also was significantly increased. Invasion of tachyzoites into mouse macrophages was significantly inhibited in the anti-rTgEF-1α PcAb pretreated group. Mice vaccinated with rTgEF-1α induced a high level of specific anti-*T. gondii* antibodies and production of IFN-gamma, interleukin-4. The expression levels of MHC-I and MHC-II molecules as well as the percentages of CD4^+^ and CD8^+^ T cells in mice vaccinated with rTgEF-1α was significantly increased, respectively (*P* < 0.05), compared with all the controls. Immunization with rTgEF-1α significantly (*P* < 0.05) prolonged survival time (14.53 ± 1.72 days) after challenge infection with the virulent *T. gondii* RH strain. These results indicate that *T. gondii* EF-1α plays an essential role in mediating host cell invasion by the parasite and, as such, could be a candidate vaccine antigen against *toxoplasmosis*.

## Introduction

*Toxoplasma gondii*, an obligate intracellular parasite, can infect virtually all warm-blooded vertebrates including wild mammals, birds, livestock, poultry, and human beings throughout the world, posing a significant public health concern (Zhao et al., 2012; Dubey et al., 2014; Zhang et al., 2014). The symptoms of *T. gondii* infection in humans ranged from asymptomatic in immunocompetent individuals to devastating in immune-compromised individuals and unprotected fetuses (Innes, 2010; Luma et al., 2013). Epidemiologic survey results showed that the high *T. gondii* prevalence in many economic animals led to considerable economic losses (Kim et al., 2009; Rassouli et al., 2011; Chessa et al., 2014).

In general, elongation factor 1-alpha (EF-1α) is highly conserved and ubiquitously expressed in all eukaryotic cells (Parkhill et al., 2000; Strausberg et al., 2002; Kristensen et al., 2005). Functionally, EF-1α transfers aminoacylated tRNAs to the ribosome A site in a GTP-dependent reaction (Riis et al., 1990). In addition, EF-1α appears to have a number of other functions associated with cell growth, motility, protein turnover, and signal transduction (Ridgley et al., 1996), more recently DNA replication/repair protein networks (Toueille et al., 2007) and apoptosis (Lamberti et al., 2007).

EF-1α has been studied in the context of pathogenicity or virulence for various microbes (Granato et al., 2004; Skarin et al., 2011; Matsubayashi et al., 2013). In the probiotic bacterium *Lactobacillus johnsonii*, the attachment to epithelial cells in the gastrointestinal tract is mediated by surface-associated EF-Tu, the prokaryotic homologue to eukaryotic EF-1α (Granato et al., 2004). The *Giardia* EF-1α protein localizes to the nuclear region in trophozoites but it relocalizes to the cytoplasm during host-cell interaction. It was a member of the excretory-secretory products of *Giardia* intestinalis trophozoites, have been suggested to be important during *Giardia* infections (Skarin et al., 2011). *Cryptosporidium parvum* (*C. parvum*) EF-1α protein, which localizes to the apical region of the parasite, mediates cryptosporidial cytoskeletal complex formation. The anti EF-1α mAb significantly inhibited *in vitro* host cell invasion by *C. parvum*. These results indicate that *C. parvum* EF-1α plays an essential role in mediating host cell entry by the parasite and, as such, could be a candidate vaccine antigen against cryptosporidiosis (Matsubayashi et al., 2013).

In this study, the full-length cDNA encoding TgEF-1α was cloned and expressed in *E. coli*. The protective efficacy induced by the recombinant TgEF-1α was evaluated, and the relevant immuno-mechanisms were investigated. This study provides the basis for the potential evaluation of TgEF-1α as a vaccine candidate or drug target against *T. gondii* infection in the future.

## Materials and Methods

### Ethics Statement

The study was approved by the Animal Care and Use Committee of Nanjing Agricultural University, in compliance with the Regulations for the Administration of Affairs Concerning Experimental Animals (The State Science and Technology Commission of China, 1988).

### Cell Culture, Animals, and Parasite

The Ana-1 mouse macrophage cell line, which were obtained from the Institute of Cell Biology, Chinese Academy Sciences (Shanghai, China), were cultured in RPMI 1640 medium containing 10% heat inactivated fetal bovine serum (FBS), 100U/ml penicillin and 100 mg/ml streptomycin at 37°C in a 5% CO_2_ atmosphere.

Five-week-old female BALB/c mice were purchased from the Center of Comparative Medicine, Yangzhou University (Yangzhou, China) and maintained under specific-pathogen-free standard conditions. All the animal experiments were approved by the Animal Ethics Committee of Nanjing Agricultural University (Approval number 200709005). *T. gondii* RH strain (Type I) was provided by Laboratory of Veterinary Molecular and Immunological Parasitology, Nanjing Agricultural University, China. To maintain the parasite, as described previously (Wang et al., 2014), BALB/c mice were intraperitoneally (i.p) injected with the parasite tachyzoites. Every 3 days, the tachyzoites were harvested and recovered from peritoneal washings of infected mice to be used for re-infection.

### Cloning and Molecular Characterization of TgEF-1α

According to the manufacturer’s protocol, Trizol reagent (Takara, Dalian, China), was used to extract total RNA from the tachyzoites of *T. gondii* RH strain and the cDNA was constructed. Primers were designed according to the nucleotide sequences of the clone *T. gondii* elongation factor 1-alpha (GenBank accession no. XM_002370208.1). The forward and reverse primers, 5′- CGCGGATCC ATGGGTAAGGAAAAGACTCACATTAAC-3′ and 5′- CCGCTCGAGCGAAGCG GTAGATTTGTTCCAAT-3′, were used to amplify the complete open reading frame (ORF) of TgEF-1α using the cDNA of *T. gondii* RH tachyzoites as a template. The underlined sequences represent the sites of BamHI and XhoI (Takara, Dalian, China), respectively. The PCR product was subcloned into the pMD19-T vector (Takara, Dalian, China) according to the manufacturer’s instructions and positive clones were selected for sequencing.

### Expression and Purification of Recombinant TgEF-1α (rTgEF-1α)

The cloned gene fragments were excised by dual BamHI/XhoI digestion and subcloned into the pET-32a (+) vectors (Novagen, USA) at the unique BamHI/XhoI site. The rTgEF-1α was expressed in the *E. coli* BL21(DE3) strain and purified by nickel-affinity chromatography according to the manufacturer’s instructions (Novagen, USA). The purity of the eluted protein was analyzed by 12% SDS-PAGE electrophoresis and Coomassie blue staining. Before mouse or rat immunization, rTgEF-1α was dialyzed against PBS (pH 7.2) and the purified protein was quantified with the Easy Protein Quantitative Kit (TransGen Biotech, Beijing, China).

### Production of Polyclonal Antibodies Against rTgEF-1α

To generate polyclonal antibodies against rTgEF-1α, SD rats were immunized subcutaneously with either 200 μg of recombinant TgEF-1α protein or PBS (control) emulsified with an equal volume of Freund’s complete adjuvant (Sigma–Aldrich, UK). Two weeks later, booster immunizations were done using either 200 μg of recombinant TgEF-1α protein or PBS emulsified with incomplete Freund’s adjuvant (Sigma–Aldrich, UK). Second booster immunizations were done 2 weeks later. Ten days after the second booster immunizations, rats were sacrificed and blood collected by cardiac puncture. Sera were extracted and affinity purified to monospecificity by affinity column chromatography as previously described (Witola et al., 2006).

### Western Blot Analysis of rTgEF-1α and Native TgEF-1α

#### Analysis of rTgEF-1α

Purified rTgEF-1α was mixed with SDS loading buffer and electrophoresed on 12% SDS-PAGE gel and electro-transferred to PVDF (Immobilon, Millipore, USA) with a Trans-Blot SD system (Bio-Rad). Non-specific binding sites were blocked by immersing the membranes in blocking buffer (5% skim milk in Tris-buffered saline containing 0.1% Tween-20) for 2 h at 37°C. The membranes were then washed five times (5 min each) in Tris-buffered saline containing 0.1% Tween-20 (TBST). Subsequently, the membranes were incubated with the primary antibody (antiserum from chickens experimentally infected with *T. gondii*) overnight at 4°C (dilutions 1:100 in TBST). After being washed five times in TBST, the membranes were then incubated with HRP-conjugated goat anti-chicken IgG (SouthernBiotech,USA) for 1 h at 37°C (diluted 1:6000 in TBST). Finally, the immunoreaction was visualized using freshly prepared diaminobenzidine (DAB, Sigma) as a chromogenic substrate after 5 min.

#### Detection of Native TgEF-1α

*Toxoplasma gondii* lysates were prepared as previously described (Hassan et al., 2014b), with minor modifications regarding this current experiment. Briefly, tachyzoites of *T. gondii* RH strain were obtained from the peritoneal washings of infected mice. The exudates were passed twice through a 27-gage needle and then through 5 μm filter membranes to remove debris and host cells. Parasites were then washed, re-suspended in phosphate-buffered saline (pH 7.4), and disrupted by sonication on ice. The lysates were electrophoresed in 12% SDS-PAGE gel and western blot analysis was performed as described in Section “Analysis of rTgEF-1”, using rat anti-rTgEF-1α polyclonal antibodies as the primary antibody and goat anti-rat IgG (SouthernBiotech, USA) as the secondary antibody.

### Passive Immunization of Mice

Mice (10 mice per group) received an intraperitoneally injection of 200 μl of the purified anti-rTgEF-1α PcAb with different concentrations (100, 200, or 500 μg/ml), or rat normal serum, or PBS alone was considerd as control. All mice were immunized intraperitoneally twice a week, total four times. Seven days after the last immunization, mice were infected intraperitoneally with 1 × 10^4^ RH strain tachyzoites. The day of infection was referred to as day 0, and the survival periods were recorded daily until all mice were dead.

### Pretreatment of *T. gondii* Tachyzoites with Anti-rTgEF-1α Polyclonal Antibodies

*Toxoplasma gondii* tachyzoites were suspended at a concentration of 4 × 10^7^/ml in RPMI 1640 containing 10% fetal bovine serum (FBS) and antibiotics. Aliquots of 100μl tachyzoites were mixed with 100 μl of the purified anti-rTgEF-1α PcAb with different concentrations (100, 200 or 500 μg/ml), or rat normal serum, or RPMI 1640 alone, respectively, and incubated at 37°C for 30 min on a platform rocker at 10 rpm. After incubation, the parasites were collected and suspended to 1 ml with RPMI 1640 medium without FBS.

#### Pretreated Tachyzoite Challenge of Mice

Mice were challenged by intraperitoneal injection of 1 × 10^4^ RH strain tachyzoites, which were pretreated with either purified anti-rTgEF-1α PcAb, rat normal serum or RPMI 1640. The day of infection was referred to as day 0, and the survival periods were recorded daily until all mice were dead.

#### Pretreated Tachyzoite Challenge of Macrophage Monolayers

Macrophage monolayers on coverslips were infected with pretreated tachyzoites (macrophage: tachyzoite = 1:5), and incubated for 4 h at 5% CO_2_ and 37°C. After 4 h, the cells were rinsed with HBSS to remove non-ingested parasites and added complete culture medium to the cells. The coverslips were then incubated for another 24 h at 5% CO_2_ and 37°C, and then fixed in methanol, and stained with Giemsa to determine the number of infected cells per 100 macrophages and number of tachyzoites/100 macrophage on light microscope.

### Evaluating the Protective Efficacy of Recombinant TgEF-1α

#### Mouse Vaccination and Challenge

A total of 120 BALB/c mice were randomly divided into four groups (30/group), and each mouse per group subcutaneously injected with either 100 μg of recombinant protein TgEF-1α mixed with Freund adjuvant (1:1), Freund adjuvant alone, or PBS only, respectively, and one group of mice was not inoculated, which served as a blank control. All groups were vaccinated three times at weeks 0, 2, and 4, respectively (the first time with Freund’s complete adjuvant and the other times with Freund’s incomplete adjuvant). The blood of mice in each group was collected on weeks 0, 2, 4 and 6 and the sera were stored at -20°C for antibodies evaluation and cytokine measurement. Two weeks after the last vaccination, the mice in the four groups were challenged intraperitoneally with 1 × 10^4^ tachyzoites of *T. gondii* RH strain. The survival time of the mice were observed and recorded on a daily basis.

#### Determination of Antibodies by enzyme-linked immunosorbent assays (ELISA)

The levels of antibodies in mouse sera were determined by enzyme-linked immunosorbent assays (ELISA) as previously described (Hassan et al., 2014a). In brief, the microtiter plates (Costar, USA) were coated with 1 μg soluble tachyzoite antigens (STAg) in 50 mM carbonate buffer (pH 9.6) and incubated at 4°C overnight. After three washes, the plates were blocked with 3% Bovine Serum Albumin (BSA) for 2 h at 37°C and subsequently incubated with the mouse sera diluted 1:10 in PBS for 1 h at 37°C. HRP-conjugated goat anti-mouse IgG, IgG1, IgG2a, IgA, IgM, and IgE, (SouthernBiotech, USA) were used as the secondary antibody to detect bound antibodies. Finally, the immune complexes were developed by incubating with 3,3,5,5-tetramethylbenzidine (TMB) for 20 min. The reaction was stopped by adding 2 M H_2_SO_4_, and the absorbance was measured at 450 nm with an automated ELISA reader (MULTISKAN FC, Thermo scientific), all samples were run in triplicate.

#### Cytokine Assays

To assay cytokine production levels, sera from each experimental group were obtained as described previously. Interferon gamma (IFN-γ), interleukin-4 (IL-4), interleukin-17 (IL-17), and transformation growth factor-β1 (TGF-β1) were measured using ready ELISA kits according to the manufacturer’s instructions (Boster Systems, Wuhan, China). Cytokine concentrations were determined by reference to standard curves constructed with known amounts of mouse recombinant IL-4, IL-17, IFN-γ, and TGF-β1. The analysis was performed with the data from three independent experiments.

#### Flow Cytometry Analysis of T Cell Subsets and MHC Molecules

The percentages of T cells subsets CD4^+^ and CD8^+^, beside MHC-I and MHC-II molecules in the splenocytes of mice in the test groups, rTgEF-1α, adjuvant, PBS, and blank, were determined using flow cytometry technique as described previously (Sasai et al., 2000). Splenocytes suspensions (1 × 10^6^ cells/ml) were dually stained with anti-mouse CD3e-FITC+anti-mouse CD8-PE, anti-mouse CD3e-FITC+anti- mouse CD4-PE, anti-mouse CD3e-FITC+anti-mouse MHC-I-PE or anti-mouse CD3e-FITC+anti- mouse MHC-II-PE (eBioscience) for 30min at 4°C in the dark. Cell population analysis was conducted by FACScan flow cytometer with CellQuest software (BD Biosciences, Franklin Lakes, NJ, USA). A lymphocyte specific gating was set according to forward and side scatters profiles. The percentages of CD4^+^ and CD8^+^ T lymphocytes, MHC-I and MHC-II molecules in mice splenocytes were determined as described by Song et al. (2010).

#### Statistical Analysis

All statistical analyses were performed by IBM SPSS 20.0 Data Editor (SPSS Inc., Chicago, IL, USA). The differences of the data (e.g., antibody responses, cytokine production) between all the groups were compared by one-way ANOVA. Survival time for the mice were compared using the Kaplan–Meier method. The results in comparisons between groups were considered different if the *P*-value was less than 0.05.

## Results

### Cloning, Expression, and Purification of Recombinant TgEF-1α

The ORF of TgEF-1α was 1347 bp encoding a protein of 448 amino acids with a predicted molecular weight of 49.04 kDa and an isoelectric point of 8.57.

**FIGURE 1:**
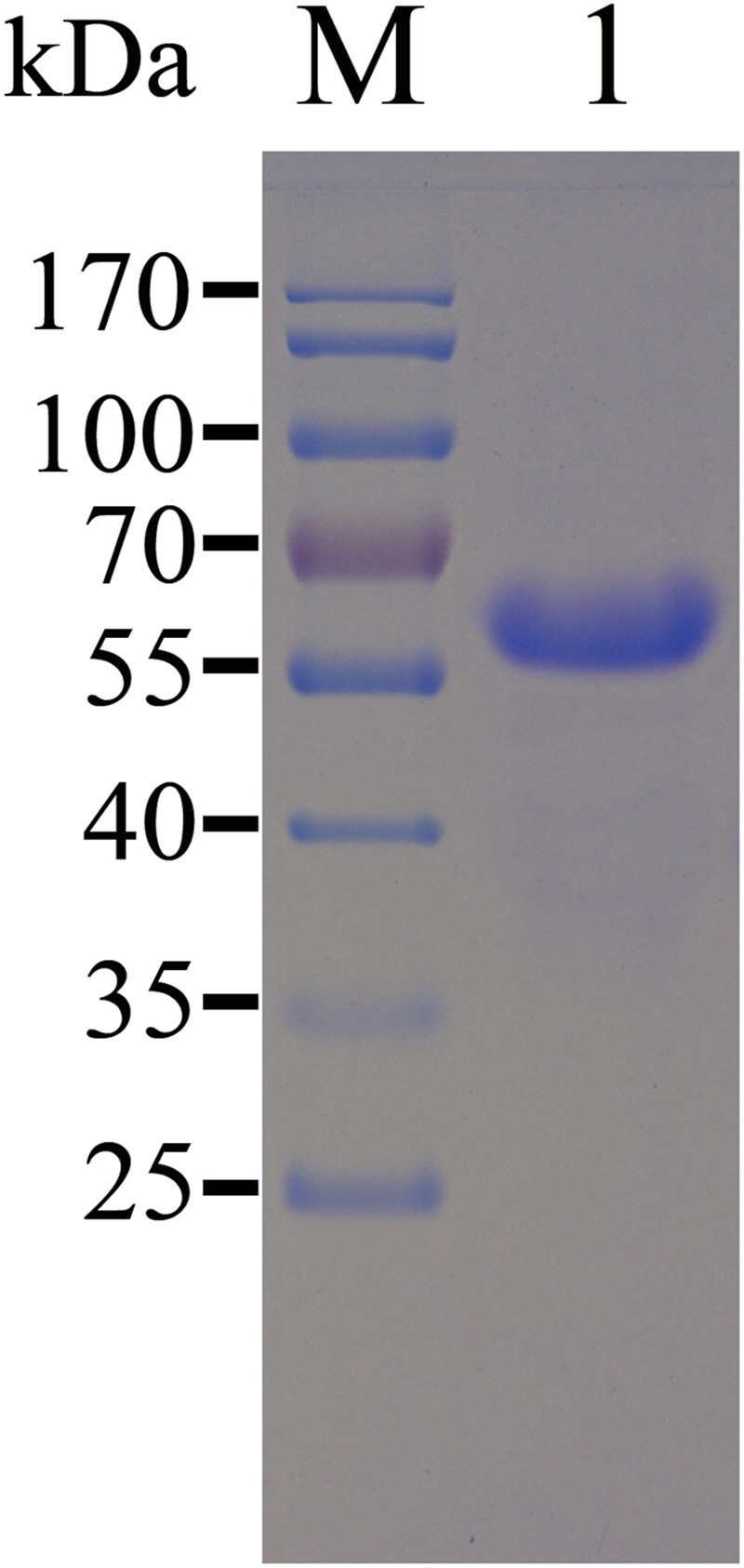
**SDS-PAGE analysis of the purification of recombinant TgEF-1α**. M: protein molecular weight marker; 1: rTgEF-1α purified through Ni2+-charged column chromatography and after dialysis.

Most of the recombinant TgEF-1α was expressed in *E. coli* as a soluble His-tagged fusion protein when bacterial growth occurred at 37°C. The soluble recombinant protein was purified by Niaffinity chromatography. SDS-PAGE analysis revealed that the recombinant protein rTgEF-1α had a molecular weight of approximately 64 kDa (**Figure 1**).

### Western Blot

**FIGURE 2:**
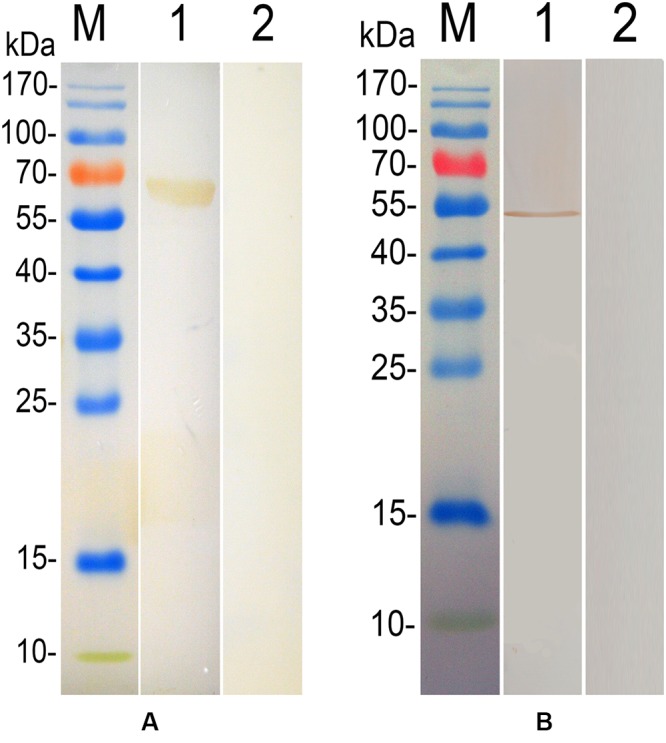
**Immunoblot for the recombinant TgEF-1α and native protein of TgEF-1α**. Lane M: standard protein molecular weight marker; **(A)** lane 1: Rrecombinant protein TgEF-1α probed by serum from chickens experimentally infected with *Toxoplasma gondii* as primary antibody; lane 2: Rrecombinant protein TgEF-1α probed by serum of normal chickens as primary antibody. **(B)** Lane 1: Sonatic extract of *T. gondii* tachyzoites probed by rat anti-rEF-1α antisera as primary antibody; lane 2: Sonatic extract of *T. gondii* tachyzoites probed by serum of normal rat as primary antibody.

Chicken anti-*T. gondii* antibodies were used to detected the fusion protein (**Figure 2A**), while a negative control experiment was also carried out using sera collected from PBS immunization group, and no specific band was detected (**Figure 2A**). The native TgEF-1α protein in lysates of *T. gondii* tachyzoites was also detected using anti-rTgEF-1α polyclonal antibodies showing a specific band (**Figure 2B**).

### Passive Immunization with Anti-rTgEF-1α PcAb Significantly Increased the Survival of Mice

Mice of PBS control group survived 6.33 ± 0.61 days after lethal tachyzoite challenge. As shown in **Table 1**, mice immunized with anti-rTgEF-1α PcAb survived significantly longer compared to the PBS control group (500 μg/ml, *p* = 0.001; 200 μg/ml, *p* = 0.001; 100 μg/ml, *p* = 0.024), while anti-rTgEF-1α PcAb (100 μg/ml) immunized mice had the longest survival time (9.46 ± 3.99 days).

**Table 1:** Survival days of BALB/c mice challenged intraperitoneally with lethal doses of tachyzoites after passive immunization with anti-rTgEF-1α antibodies.

Group	Survival days^a^	*p*-value^b^	Survival rate (%)
PBS control	6.33 ± 0.61	–	0
Serum control	6.70 ± 0.48	0.155	0
Anti- rTgEF-1α-A	8.56 ± 1.53	0.001	0
Anti- rTgEF-1α-B	8.80 ± 1.75	0.001	0
Anti- rTgEF-1α-C	9.46 ± 3.99	0.024	10

*Anti- rTgEF-1 α-A: mice received i.p. injection of 200 μl of the purified anti-rTgEF-1α PcAb (500 μg/ml); Anti- rTgEF-1 α-B: mice received i.p. injection of 200 μl of the purified anti-rTgEF-1α PcAb (200 μg/ml); Anti- rTgEF-1 α-C: mice received i.p. injection of 200 μl of the purified anti-rTgEF-1α PcAb (100 μg/ml);*

^a^*Mice were observed 20 days. The data are presented as the mean ± SD of three separate experiments*.

^b^*Compared to the survival time of PBS control group*.

### Immunization with Tachyzoites Pretreated with Anti-rTgEF-1α PcAb Significantly Increased the Survival of Mice

*Toxoplasma gondii* tachyzoites were pretreated with anti-rTgEF-1α PcAb following the protocol, and were then injected into mice. As shown in **Table 2**, the survival time of the RPMI 1640 control and serum control group were 6.33 ± 0.61 and 6.53 ± 0.63 days, respectively. Mice that received anti-rTgEF-1α PcAb (200 μg/ml and 100 μg/ml) pretreated tachyzoites survived significantly longer than those that received RPMI 1640 (*p* < 0.05), control serum pretreated tachyzoites (*p* < 0.05). However, pretreatment with anti-rTgEF-1α PcAb (500 μg/ml) did not produce significantly longer survival times than RPMI 1640 (*p* > 0.05) and serum control group (*p* > 0.05).

**Table 2:** Survival days of BALB/c mice challenged intraperitoneally with lethal doses of tachyzoites pretreated with anti-rTgEF-1α antibodies.

Group	Survival days^a^	*p*-value^b^	Survival rate (%)
RPMI 1640 control	6.33 ± 0.61	–	0
Serum control	6.53 ± 0.63	0.483	0
Anti-rTgEF-1α-A	7.30 ± 1.47	0.071	0
Anti-rTgEF-1α-B	9.66 ± 3.93	0.016	10
Anti-rTgEF-1α-C	8.53 ± 1.78	0.009	0

*Anti- rTgEF-1 α-A: mice received i.p. injection of *T. gondii* tachyzoites pretreated with anti-rTgEF-1α PcAb (500 μg/ml); Anti- rTgEF-1 α-B: mice received i.p. injection of *T. gondii* tachyzoites pretreated with anti-rTgEF-1α PcAb (200 μg/ml); Anti- rTgEF-1 α-C: mice received i.p. injection of *T. gondii* tachyzoites pretreated with anti-rTgEF-1α PcAb (100 μg/ml);*

^a^*Mice were observed 20 days. The data are presented as the mean ± SD of three separate experiments*.

^b^*Compared to the survival time of RPMI 1640 control group*.

### Anti-rTgEF-1α PcAb Pretreatment Inhibited Parasite Invasion in Macrophages

The percentage of infected Ana-1 macrophages in RPMI 1640 control was 63.33 ± 8.62%. There were slightly decreased the mean percentage of infected macrophages after pretreatment with control serum (*p* > 0.05). As shown in **Table 3**, pretreatment of tachyzoites with anti-rTgEF-1α PcAb significantly inhibited parasite invasion in macrophages compared to the a RPMI 1640 control group (63.33 ± 8.62 vs. 36.33 ± 6.81 in 500 μg/ml anti-rTgEF-1α group, *p* < 0.05; 63.33 ± 8.62 vs. 34.67 ± 5.51 in 200 μg/ml anti-rTgEF-1α group, *p* < 0.01; 63.33 ± 8.62 vs. 35.67 ± 5.69 in 100 μg/ml anti-rTgEF-1α group, *p* < 0.05). Similarly, there were significant differences in the number of intracellular parasites per macrophage between PcAb treated and untreated groups (*p* < 0.01).

**Table 3:** Macrophage invasion and multiplication of tachyzoites after infection by *T. gondii* pretreated with anti-rTgEF-1α antibodies.

Group	No.of infected cells/100 Mac	*p*-value^a^	No. of tachyzoites/ cell	*p*-value^b^
RPMI 1640 control	63.33 ± 8.62	–	6.70 ± 0.36	–
Serum control	57.00 ± 4.58	0.324	5.93 ± 0.42	0.073
Anti-rTgEF-1α-A	36.33 ± 6.811	0.013	4.83 ± 0.38	0.003
Anti-rTgEF-1α-B	34.67 ± 5.51	0.008	4.77 ± 0.46	0.005
Anti-rTgEF-1α-C	35.67 ± 5.69	0.010	4.73 ± 0.40	0.003

*Anti- rTgEF-1 α-A: macrophage received *T. gondii* tachyzoites pretreated with anti-rTgEF-1α PcAb (500 μg/ml); Anti- rTgEF-1 α-B: macrophage received *T. gondii* tachyzoites pretreated with anti-rTgEF-1α PcAb (200 μg/ml); Anti- rTgEF-1 α-C: macrophage received *T. gondii* tachyzoites pretreated with anti-rTgEF-1α PcAb (100 μg/ml);*

^a^*Compared to the RPMI 1640 control group*.

^b^*Compared to the RPMI 1640 control group*.

### Humoral Immune Responses Induced by Vaccination

**FIGURE 3:**
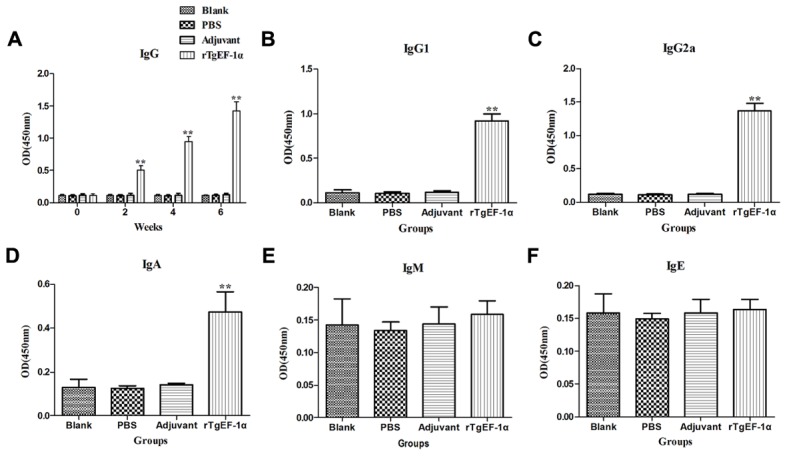
**The dynamics of specific antibody level in BALB/c mice induced by rTgEF-1α vaccination**. **(A)** Determination of IgG antibodies in the sera of BALB/c mice immunized with rTgEF-1α, Adjuvant, PBS and Blank controls on weeks 0, 2, 4, 6. Determination of IgG subclass **(B)** IgG_1_ and **(C)** IgG_2a_, **(D)** levels of class IgA, **(E)** levels of class IgM, and **(F)** levels of class IgE in the sera of the immunized BALB/c mice 2 weeks after the last immunization. Results are expressed as mean of the OD450 ± SD (*n* = 5) and statistically significant difference (^∗^*P* < 0.05) and (^∗∗^*P* < 0.01).

To detect the levels of anti-*T. gondii* antibodies, all sera were tested by ELISA. As shown in **Figure 3A**, a significantly higher levels of IgG antibodies were detected in the sera of mice immunized with rTgEF-1a (*P* < 0.01) versus control groups and the levels of antibodies increased with successive immunizations. As expected, no augmentation in antibody levels was detected in the control mice (**Figure 3A**). Moreover, both IgG1 and IgG2a were found in the sera of mice vaccinated with rTgEF-1a, which showed a mixed anti-*T. gondii* IgG1/IgG2a profile (**Figures 3B,C**). A predominance of IgG2a over IgG1 was observed in the sera of mice immunized with rTgEF-1a, which indicated a shift toward the Th1 type response.

In comparison to the control groups, dynamics of the IgA demonstrated high OD values (*P* < 0.01) in the immunized group (0.473 ± 0.093) at two weeks after last immunization (**Figure 3D**). However, IgM and IgE activity showed no significant changes at the time of evaluation (**Figures 3E,F**).

### Cytokine Measurement

**FIGURE 4:**
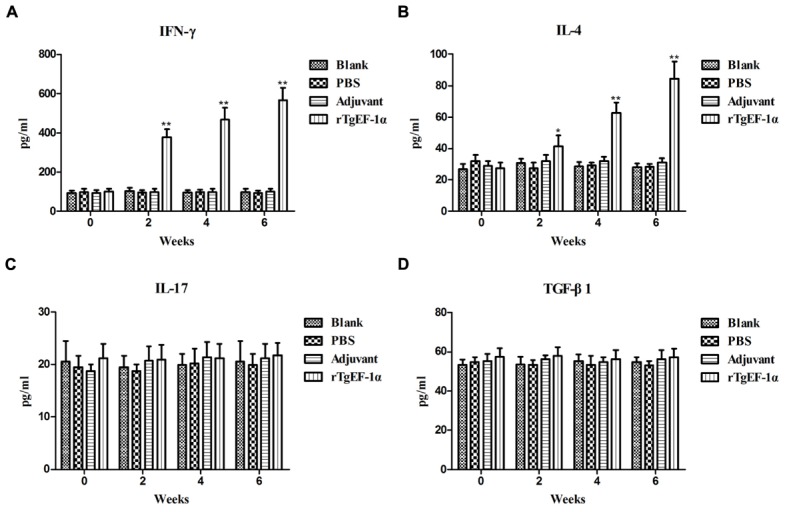
**The dynamics of cytokine production in BALB/c mice induced by rTgEF-1α vaccination**. Antibody-captured ELISA was used to determine the production levels of **(A)** IFN-γ, **(B)** IL-4, **(C)** IL-17, and **(D)** TGF-β1, in sera samples (*n* = 5) collected at weeks 0, 2, 4, and 6, and the comparison results were expressed as means ± SD of pg/ml. The asterisk designates statistically significant differences (^∗^*p* < 0.05; ^∗∗^*p* < 0.01) between groups. Results presented here were from three independent experiments.

Significantly higher levels of IFN-γ were produced in the sera of mice immunized with rTgEF-1α compared with the three control groups (*P* < 0.01, **Figure 4A**). On the other hand, low levels of IL-4 were detected in the groups of mice immunized with rTgEF-1α, which showed a slight but significant difference (*P* < 0.05) compared with mice immunized with the three control groups (**Figure 4B**). As for IL-17 and TGF-β1, both of them displayed no significant changes at similar times of evaluation (**Figures 4C,D**).

### Evalution of the Percentages of CD4^+^ and CD8^+^ T Lymphocytes and MHC Molecule Changes

The percentage of CD4^+^ lymphocyte were found to be increased significantly in the spleen lymphocytes of mice immunized with rTgEF-1a at week 2, 4, and 6 when compared with with mice immunized with the three control groups (*P* < 0.01). The percentage of CD8^+^ lymphocyte also were found to be increased significantly in the spleen lymphocytes of mice immunized with rTgEF-1a at week 4 and 6 when compared with mice immunized with the three control groups (*P* < 0.05, **Table 4**).

**Table 4:** Flow cytometry analysis of the percentages of T lymphocyte subsets.

Marker (%)	Time point	Groups (*n* = 5)			
		Blank	PBS	Adjuvant	rTgEF-1α
CD4^+^	Week 0	18.01 ± 2.95	18.53 ± 2.76	18.15 ± 2.71	18.18 ± 2.36
	Week 2	18.41 ± 1.85	18.55 ± 1.96	18.30 ± 2.78	25.14 ± 2.87^∗∗^
	Week 4	18.03 ± 2.84	18.80 ± 2.88	18.24 ± 1.93	28.27 ± 2.98^∗∗^
	Week 6	18.17 ± 1.54	17.93 ± 2.31	18.68 ± 2.62	30.46 ± 3.43^∗∗^
CD8^+^	Week 0	8.20 ± 1.55	8.13 ± 1.28	8.29 ± 1.63	8.36 ± 1.76
	Week 2	8.32 ± 1.94	8.00 ± 1.48	8.54 ± 1.15	9.17 ± 1.84
	Week 4	8.37 ± 2.07	8.13 ± 1.58	8.45 ± 1.89	11.53 ± 2.91^∗^
	Week 6	8.11 ± 1.12	8.01 ± 1.54	8.24 ± 1.57	12.36 ± 1.47^∗∗^

*Data are presented as the mean ± SD (n = 5). ^∗∗^represents statistically highly significant difference (*P* < 0.01) as compared with control groups: Blank (unvaccinated control), PBS and Adjuvant group. ^∗^represents statistically highly significant difference (*P* < 0.05) as compared with control groups: Blank (unvaccinated control), PBS and Adjuvant group.*

After the last immunization, MHC-I molecules of immunized group showed high significant readings (30.47 ± 3.15), in contrast to adjuvant (18.34 ± 2.40), PBS (17.76 ± 1.16), and blank (17.88 ± 1.78) groups. Concerning MHC-II molecules, an increasing pattern was noticed in the vaccinated group starting at week 4 of the experiment and reaching a peak point (7.96 ± 1.37) at week 6 of the experiment (**Table 5**).

**Table 5:** Dynamics of MHC-I and MHC-II molecules in spleen lymphocytes.

Marker (%)	Time point	Groups (*n* = 5)			
		Blank	PBS	Adjuvant	rTgEF-1α
MHC-I	Week 0	18.19 ± 2.46	17.17 ± 2.62	18.06 ± 1.54	18.01 ± 2.23
	Week 2	16.55 ± 2.15	17.13 ± 1.91	18.17 ± 2.31	24.28 ± 3.29^∗∗^
	Week 4	17.64 ± 1.82	18.02 ± 1.91	18.22 ± 1.76	29.16 ± 3.43^∗∗^
	Week 6	17.88 ± 1.78	17.76 ± 1.16	18.34 ± 2.40	30.47 ± 3.15^∗∗^
MHC-II	Week 0	3.07 ± 0.97	2.88 ± 0.37	3.22 ± 0.62	3.16 ± 0.54
	Week 2	2.99 ± 0.71	3.17 ± 0.38	3.13 ± 0.79	3.44 ± 0.96
	Week 4	3.25 ± 0.45	3.17 ± 0.38	3.04 ± 0.46	6.78 ± 1.48^∗∗^
	Week 6	3.22 ± 0.56	3.11 ± 0.73	3.05 ± 0.61	7.96 ± 1.37^∗∗^

*Data are presented as the mean ± SD (*n* = 5). ^∗∗^represents statistically highly significant difference (*P* < 0.01) as compared with control groups: Blank (unvaccinated control), PBS and Adjuvant group*.

### Protective Efficacy of rTgEF-1a in BALB/c Mice

**FIGURE 5:**
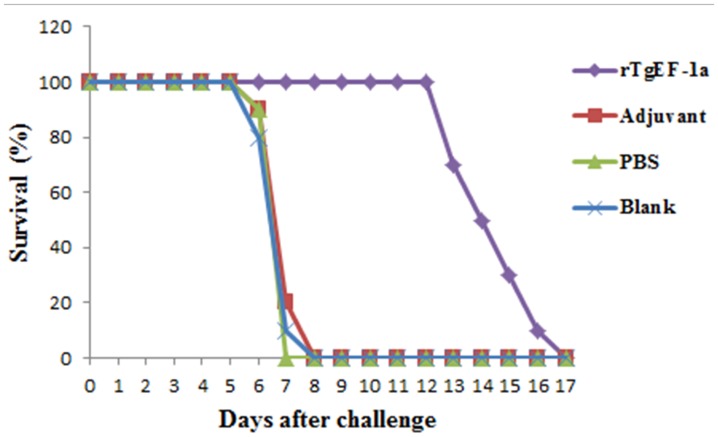
**Survival curve of mice after challenge infection with *T. gondii* RH strain**. Mice were challenged with 10^4^ tachyozoites of the RH strain intraperitoneally two weeks after the third immunization.

Survival curves for the four groups of mice are shown in **Figure 5**. No differences were observed among the three control groups, all of the mice in control groups died within 8 days post-challenge. Mice immunized with rTgEF-1a showed a prolonged survival time (14.53 ± 1.72 days; *P* < 0.01) compared to the three control groups.

## Discussion

In this study, we compared the protective effect of passive immunization with PcAbs to TgEF-1a at different concentrations. The period of survival days was significantly increased in mice passively immunization with PcAb to TgEF-1a compared to the serum and PBS control group. Similarly, mice immunized with anti-SAG1 mAb, anti-GRA2 mAb, or anti-GRA6 mAb survived longer than mice treated with control ascites (Mineo and Kasper, 1994; Cha et al., 2001; Fu et al., 2011). Another passive immunization experiment using monoclonal antibodies against nucleoside triphosphate hydrolase-II (NTPase-II) showed no inhibition of parasite invasion, but a significant reduction in *T. gondii* replication and promoted prolonged survival in mice after a lethal challenge (Tan et al., 2010). From these results, it appears that PcAb to TgEF-1a protein, as well as mAbs to NTPase-II, SAG1 or GRA proteins of *T. gondii*, are capable of providing protection in the absence of an already developed cell-mediated immunity.

Interesting, mice survived better when immunized passively with lower antibody concentration. In addition, Mice that received anti-rTgEF-1α PcAb (200 μg/ml or 100 μg/ml) pretreated tachyzoites survived better than that received tachyzoites pretreated with anti-rTgEF-1α PcAb (500 μg/ml). The reason should be further studied.

Host cell invasion by tachyzoites plays a crucial role in maintaining *T. gondii* infection. Many factors involved in host cell invasion or intracellular multiplication had been reported (Mineo and Kasper, 1994; Huynh and Carruthers, 2006; Dunn et al., 2008; Beck et al., 2013; Huynh et al., 2014). In this study, we directly evaluated the inhibitory effect of cell invasion by anti-TgEF-1a PcAb using mouse macrophages. Anti-TgEF-1a PcAb pretreated tachyzoites exhibited inhibition of parasite invasion into mouse macrophage compared to the control groups. Additionly, mice that received anti-rTgEF-1α PcAb (200 μg/ml and 100 μg/ml) pretreated tachyzoites survived significantly longer than those that received RPMI 1640, control serum pretreated tachyzoites (*p* < 0.05). Taken together, anti-TgEF-1a PcAb could inhibit the parasite invasion into mouse macrophage cell line as well as mouse model. These results indicated that TgEF-1α played an essential role in mediating host cell invasion. Further analysis is needed to clarify the molecular role of TgEF-1α during parasite attachment and invasion of host cells.

The critical role of antibody in immunity to *T. gondii* has been recognized for a long time, referred to the ability of killing the parasite by the attachment of the parasite to the host cell receptors or resulting from the bindings to the complement protein (Correa et al., 2007). The mice immunized with rTgEF-1α developed higher levels of *T. gondii*-specific IgG antibodies compared to mice of control groups. Meanwhile, vaccination with rTgEF-1α exhibited a mixed Th1/Th2 response, with a predominance of IgG2a (Th1) over IgG1 (Th2). Many studies demonstrated that a Th1-biased response is required for effective protection against naturally occurring *T. gondii* infections (Sayles et al., 2000).

In our study, high titers of IgA were detected in the sera of rTgEF-1α immunized group, indicating that rTgEF-1α has successfully induced the release of this antibody as part of the response generated after immunization. Immunoglobulins IgM and IgE aslo were reported to participate in the immunological responses against *T. gondii* infection (Gomez-Marin et al., 2000; Lynch et al., 2011; Vouldoukis et al., 2011; Amin et al., 2012). However, our data revealed no significant changes of these two immunoglobulin after vaccination with rTgEF-1α.

During natural *T. gondii* invasion, the generation of strong cellular immune responses determines the course of the infection (Dupont et al., 2012; Hotop et al., 2014). In contrast to three control groups, immunization with rTgEF-1α enhanced the Th1 mediated immunity with high levels of IFN-γ. The cytokine IL-4 is associated with Th2-type responses, and a slight increase in the release of IL-4 was also observed in the present study. Therefore, these findings indicated that specific Th1-type cell immune response was mainly activated after immunization with rTgEF-1α.

Due to the obligate intracellular life style, T cell mediated adaptive immune responses involving in CD4^+^ and CD8^+^ T cells are known to be important in resistance against primary *T. gondii* infection and reactivation of chronic toxoplasmosis (Kugler et al., 2013; Dupont et al., 2014). In the present study, we observed the increase of both the percentage of CD4^+^ and CD8^+^ T cells in mice immunized with rTgEF-1α, which suggested the activation of CD4^+^ and CD8^+^ T cells, and thus may be in synergy to contribute to cytotoxic activity against *T. gondii*. Meanwhile, the significant increase of the MHC class I and MHC class II molecules was also found in the rTgEF-1α vaccinated group, suggesting that both the exogenous (MHC class II restricted) and the endogenous (MHC class I restricted) antigen presentation pathways were simultaneously activated. The MHC class I restricted presentation results in a strong CD8^+^ T cell mediated immune responses, which enhance the cytoloxicity against cells infected with *T. gondii.* The up-regulated MHC-II molecules could present more *T. gondii*-derived antigenic peptides to CD4^+^ T cells and induce more strong immune responses during *T. gondii* infection, leading to inhibition of the parasite (Dupont et al., 2014). Take together, the results of the rTgEF-1α immunization experiments show that the rTgEF-1α can induce strong humoral and cellular immune responses.

Two weeks after the last immunization with rTgEF-1α, lethal challenge experiments showed that rTgEF-1α could prolong survival time in BALB/c mice challenged with *T. gondii* tachyzoites (RH strain) when compared with control groups. However, all mice in our experiment were dead after challenged with tachyzoites. The current results indicated that the rTgEF-1α can only induce partial protection against infection with high virulent *T. gondii* strain but not complete, which was similar to the effect of other single gene subunit vaccines in mice (Huang et al., 2012; Zheng et al., 2013).

In conclusion, the present study demonstrated, for the first time, that TgEF-1α plays an essential role in mediating host cell entry by the parasite, and that TgEF-1α induce a strong protective humoral and cellular response against *T. gondii* infection, which indicated that it is a potential candidate vaccine antigen against toxoplasmosis. The strategy of using TgEF-1α protein combined with other antigens appears to be a promising approach to develop a new subunit multi-component vaccine against toxoplasmosis.

## Author Contributions

SW analyzed data and wrote the paper; XL designed the research; ZZ, YW, JG, LX, RY, and XS performed the experiments; XL approved the version to be published.

## Conflict of Interest Statement

The authors declare that the research was conducted in the absence of any commercial or financial relationships that could be construed as a potential conflict of interest.
